# The excitability of ipsilateral motor evoked potentials is not task-specific and spatially distinct from the contralateral motor hotspot

**DOI:** 10.1007/s00221-024-06851-6

**Published:** 2024-06-06

**Authors:** Nelly Seusing, Sebastian Strauss, Robert Fleischmann, Christina Nafz, Sergiu Groppa, Muthuraman Muthuraman, Hao Ding, Winston D. Byblow, Martin Lotze, Matthias Grothe

**Affiliations:** 1 Department of Neurology, University Medicine of Greifswald, Greifswald, Germany; 2 Imaging and Neurostimulation, Department of Neurology, University Medical Center of the Johannes Gutenberg University Mainz, Mainz, Germany; 3 Neural Engineering with Signal Analytics and Artificial Intelligence (NESA-AI), Department of Neurology, University Medicine of Würzburg, Würzburg, Germany; 4 Movement Neuroscience Laboratory, Department of Exercise Sciences, The University of Auckland, Auckland, New Zealand; 5 Centre for Brain Research, University of Auckland, Auckland, New Zealand; 6 Functional Imaging Unit, Center for Diagnostic Radiology, University Medicine Greifswald, Greifswald, Germany

**Keywords:** Human, Upper limb, Motor evoked potential primary motor cortex, Ipsilateral movements.

## Abstract

**Objective:**

The role of ipsilateral descending motor pathways in voluntary movement of humans is still a matter of debate, with partly contradictory results. The aim of our study therefore was to examine the excitability of ipsilateral motor evoked potentials (iMEPs) regarding site and the specificity for unilateral and bilateral elbow flexion extension tasks.

**Methods:**

MR-navigated transcranial magnetic stimulation mapping of the dominant hemisphere was performed in twenty healthy participants during tonic unilateral (iBB), bilateral homologous (bBB) or bilateral antagonistic elbow flexion-extension (iBB-cAE), the map center of gravity (CoG) and iMEP area from BB were obtained.

**Results:**

The map CoG of the ipsilateral BB was located more anterior-laterally than the hotspot of the contralateral BB within the primary motor cortex, with a significant difference in CoG in iBB and iBB-cAE, but not bBB compared to the hotspot for the contralateral BB (each *p* < 0.05). However, different tasks had no effect on the size of the iMEPs.

**Conclusion:**

Our data demonstrated that excitability of ipsilateral and contralateral MEP differ spatially in a task-specific manner suggesting the involvement of different motor networks within the motor cortex.

## Introduction

Voluntary limb movements are executed by the ipsilateral and contralateral primary motor cortex. Several imaging studies demonstrated that especially the ipsilateral primary and premotor cortices are activated during the coordination of bimanual movements and for maintaining posture (Bundy and Leuthardt 2019). These ipsilateral motor cortical areas are also recruited to compensate for congenital or acquired brain injury (Staudt et al., 2002; Bradnam et al. 2013).

The recruitment of ipsilateral motor pathways can be examined neurophysiologically with transcranial magnetic stimulation (TMS) (Armand and Kuypers 1980; Wassermann et al. 1994; Ziemann et al. 1999). Ipsilateral motor evoked potentials (iMEP) are distinct from the contralateral MEPs (cMEP) in a few important ways. They tend to have a later onset, a higher threshold and smaller size, indicating a weaker and possibly indirect route to the spinal alpha motor neurons. Furthermore, iMEPs can been obtained more readily in proximal compared to distal muscles (Bawa et al. 2004; Wassermann et al. 1994) and are usually only present when the target muscle is pre-activated (Bawa et al. 2004; Chen et al. 2003). These characteristics reinforce their potential importance for bimanual or postural motor interaction.

Tazoe and colleagues also described a task dependence of iMEP excitability (Tazoe and Perez 2014). In their study, iMEPs obtained from the non-dominant biceps brachii (BB) were smaller during contraction of both BB compared to unilateral, non-dominant BB activation. In addition, heterologous bilateral movements, which required contraction of the non-dominant BB with coincident contraction of the dominant triceps brachii, revealed the largest iMEPs compared to task contexts. The authors speculated this was due to a modulatory influence of interhemispheric inhibition (Perez et al. 2014) and neck afferent inputs (Tazoe and Perez 2014).

Another explanation for a task related difference in iMEPs could relate, at least in part, to a task-dependency of the cortical iMEP representation, captured by the “hotspot”. Most commonly, the hotspot for the contralateral MEP is assumed to be the hotspot for the iMEP (Chen et al. 2003; McCambridge et al. 2016; Tazoe and Perez 2014). An alternative approach is to calculate a center of gravity (CoG) from a grid of scalp locations, as has been done in somatotopic investigations (Lotze et al. 2003). However, only a few iMEP studies used CoG for describing the location of the iMEP. Ziemann et al. (1999) found a lateral and anterior shift of the CoG of the first dorsal interosseous (FDI) representation in seven participants during unilateral tonic contraction compared to the cMEP hotspot. In contrast, the CoG of the BB iMEP was more medial and anterior during tonic unilateral contraction in the 16 participants examined by Tazoe (2014). Furthermore, a difference was found between FDI iMEPs and proximal deltoid in seven participants studied by Wasserman and colleagues (Wassermann et al. 1994). During tonic unilateral contraction, FDI iMEPs were elicited for a hotspot that was more lateral, and deltoid iMEPs from a hotspot that was more medial compared to cMEPs. In most cases iMEP hotspots exhibit greater variability between participants than those used to obtain cMEPs. Partially profound methodological differences between studies further complicate the interpretation about iMEP hotspot characterization which is needed for a better understanding of ipsilateral motor pathways, and their potential modulatory use in disease conditions like stroke (Bradnam et al. 2013).

The primary aim of the present study was to investigate whether there is a task-dependent difference in ipsilateral M1 CoG compared to the contralateral CoG representation using MR navigated TMS. A secondary aim was to confirm the task specific modulation of the ipsilateral MEPs.

## Methods

### Participants

In total, twenty healthy volunteers (10f, mean 26.1 ± 4.6y) participated in the study. All participants were right handed as determined by Edinburgh Handedness inventory (Oldfield 1971); mean score 94.3). The sample size of this exploratory study was determined based on the previous studies that used samples of less than twenty participants. None of the participants took any medication or had any neurological or psychiatric disorder. All participants gave written informed consent to the experimental procedures, which were approved by the local ethics committee at the University Medicine of Greifswald (BB139/18).

### Structural MRI

A 3T scanner (Verio, Siemens, Erlangen, Germany) with a 32-channel head coil was used for MR imaging. A high-resolution T1-weighted 3D MPRAGE (voxel size 1 × 1 × 1 mm; 176 slices; matrix size 256 × 256; TR 1.69 ms; TE 2.52 ms) was generated for TMS-neuronavigation.

### Navigated- TMS

During the TMS experiment participants were seated in a comfortable chair, connected to the EMG and registered for frameless neuronavigation. Electromyographic (EMG) activity was recorded from the left and right biceps brachii using a tendon-belly-montage with surface electrodes (10 mm Ag/AgCl) Recorded EMG signals were amplified (CED 1902; Cambridge Electronic Design, United Kingdom), band-pass filtered (20–1000 Hz) and sampled at 2 kHz (CED 1401). Data were stored for offline analysis using Signal (V6.0, CED).

TMS was delivered through a Magstim Bistim 200 stimulator (MagStim Company Ltd.) with a monophasic waveform. Neuronavigation was performed with a stereo-tactical infrared optical-tracking Polaris camera (Polaris System, Northern Digital, Waterloo, Ontario, Canada) and BrainSight (BrainSight TMS, Rogue Research Inc.). The individual structural MR scan and participant head were co-registered in the Polaris reference frame using 3D-head reference marker attached to the head, with the tragi, nose tip and nasion used as anatomical landmarks. After registration, the left primary motor cortex was localized by identifying the ‘hand knob’ as an anatomical landmark for the motor hand area (Yousry et al. 1997).

### Experimental setup

The figure-eight coil was held tangentially to the scalp at an angle of 45° to induce current flow in a posterior to anterior direction. TMS was delivered starting at the anatomical landmark of the hand knob of the dominant left hemisphere. For the cMEP motor hotspot, the coil was moved until the site eliciting the largest average MEPs in the resting BB contralateral to the simulation side in 5 of 10 stimuli with an intensity of 60% maximum stimulator output (MSO) was located.

cMEP hotspot location was stored and used as the center of a 3 × 3 grid with an in-between distance of 2 cm, spanning a grid of 4 cm × 4 cm in total for each of the iMEP conditions. The aim of this grid was to create a target area covering CoG locations and to reliably stimulate the targets across the three conditions (Zdunczyk et al. 2013). The grid was created using the Brainsight build-in function and was snapped to the individual reconstructed 3D brain surface. Coordinates of the nine iMEP targets including the cMEP hotspot were stored for further offline analyses.

**Fig. 1:**
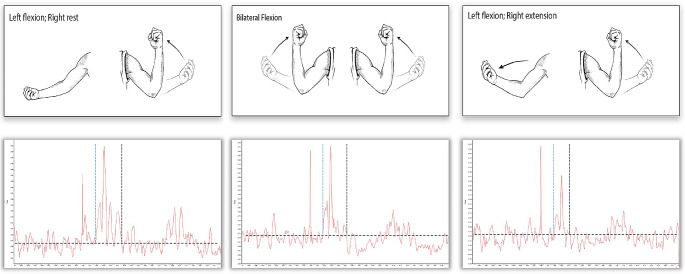
Schematic illustration of the iMEP modulation tasks: (**a**) unilateral voluntary contraction with flexion of the left biceps brachii and relaxation of the right arm, (**a**) bilateral homologous voluntary contraction with bilateral flexion of both biceps brachii, (**a**) bilateral antagonistic voluntary contraction with flexion of the left biceps brachii and extension of the right arm. Note that the tasks were isometric. The lower row shows raw, rectified EMG data of a representative subject for each condition. Horizontal dashed lines schematically represent the mean background EMG, while horizontal lines denote the onset and offset of iMEPs.

All of the determined 9 stimulation target points were stimulated in a randomized order with 10 pulses at 100% maximum stimulator output (MSO) each to guarantee reliable recordings (Cavaleri et al. 2017). The iMEP experiment consisted of three different tasks, which was performed in a counterbalanced order between the participants (see Fig. 1). Stimulation of the dominant left hemisphere was performed while:

- *unilateral contraction* (iBB): contraction of the left biceps brachii and relaxation of the right arm.
- *bilateral homologous contraction (*bBB): bilateral contraction of both biceps brachii;
- *bilateral antagonistic contraction (*iBB-cAE): contraction of the left biceps brachii and extension of the right arm.

Participants were asked to keep their head straight at all times and were encouraged to perform an isometric contraction of 50% maximal voluntary contraction in the biceps brachii ipsilateral to stimulation side. Muscle force was visually controlled using a dynamometer. Within the tasks, participants were given time to rest as needed.

### Data processing

iMEP onset was defined as timepoint when poststimulus EMG exceed prestimulus EMG by one standard deviation for at least 5ms (Ziemann et al. 1999). iMEP offset was defined as the time point where the EMG dropped below the mean rectified EMG plus one standard deviation for more than 5ms. iMEP onset and offset were determined by visual inspection using a horizontal line marking the mean of the rectified EMG before the TMS stimulus plus one standard deviation. Relative iMEP area was calculated using the following formula: [area of rectified EMG in iMEP duration / (mean prestimulus EMG*iMEP duration)*100] (Tazoe and Perez 2014). The iMEP area reflects the proportionate size of iMEP relative to the prestimulus EMG, expressed as a percentage. The prestimulus EMG was measured 100ms before the TMS stimulus. Background EMG was kept constant over different conditions.

For each of the nine stimulation sites, mean iMEP areas were calculated and ranked separately resulting in nine mean iMEP areas per task per participant.

Locations of individual iMEP-CoGs were calculated using the nine stimulation locations and respective MEP areas as

$${x}_{CoG}=\frac{{\sum }_{i}{a}_{i}{x}_{i}}{{\sum }_{i}{a}_{i}}$$

where *a* represents the response area in µV at the 3D locatxion vector *x* for position *i* of stimulation grid (Miranda et al. 1997). The CoG represents the mean location of stimulation points weighted for the amplitudes of their respective responses.

Coordinates of individual cMEP and calculated task dependent iMEP CoG were normalized to the Colin 27 MNI-brain (Montreal Neurological Institute, McGill University). Comparisons of individual cMEP and iMEP CoG location were performed with a visual approach using the coordinates superimposed on Colin 27 MNI-brain using the normalization pipeline of SPM V 12 (http://www.fil.ion.ucl.ac.uk/spm/) implemented in in-house written scripts implemented in Matlab 2016b (MathWorks Inc., Sherborn, MA).

### Statistical analysis

Individual mean iMEP area was calculated for each target point of the grid for each task separately. Levene’s test was performed to test for heteroscedasticity.

For the primary hypothesis (task dependent differences in CoG between iMEP and cMEP), a comparison of the distributions of centroids assessed by the IMEP separately (iBB, bBB, iBB-cAE) and cMEP were evaluated by Bayesian statistics. The BEST R package (https://jkkweb.sitehost.iu.edu/BEST/) for estimation of the Bayesian posterior distribution. Bayesian posterior analysis was performed using the Markov Chain Monte Carlo approach for the choice of priors; the default Markov Chain Monte Carlo sample size of 100,000 was used for all analyses. Bayesian analysis provides complete distributions of credible values for group means and their differences (Kruschke 2013). Group differences were calculated as differences in the means and are shown as differences between the groups, which describes the ability to separate the compared groups. Differences with an accuracy ≥ 80% were interpreted as significant (* *p* < 0.05). Accuracies ≥ 90% were regarded as highly significant (** *p* < 0.01), while differences above 95% between the group values were assigned the highest significance (*** *p* < 0.001). An 80% accuracy for differentiating between two groups is defined by a 20% probability of rejecting a true null hypothesis. This corresponds to a p value of *p* = 0.05 (Johnson 2013), hence our cut-off. Reporting guidelines written by the developer of the Bayesian toolbox also confirm that 80% is within the range to accept the null hypothesis with a credible effect size analyzed by Bayesian posterior probability (Kruschke 2021). This type of analyses have successfully been applied in studies with lower sample size compared to this study in previous publications (Hanuscheck et al. 2022; Michels et al. 2017).

The secondary aim was to confirm the task specific modulation of the iMEPs. Task depended shifts of iMEP CoG were presented using scatterplot visualization to drew parallels to existing literature (Ziemann et al. 1999). In addition, a linear mixed effects model (LMM) was performed with TASK (iBB, bBB, iBB-cAE) as fixed effect and subject and background EMG as random effects to assess the task-dependency of the iMEP areas. Analyses were done twice, with the maximum mean iMEP area target point per task and the iMEP area at the hotspot of the contralateral BB. Significance was set at *p* < 0.05.

## Results

### Center of gravity

**Fig. 2:**
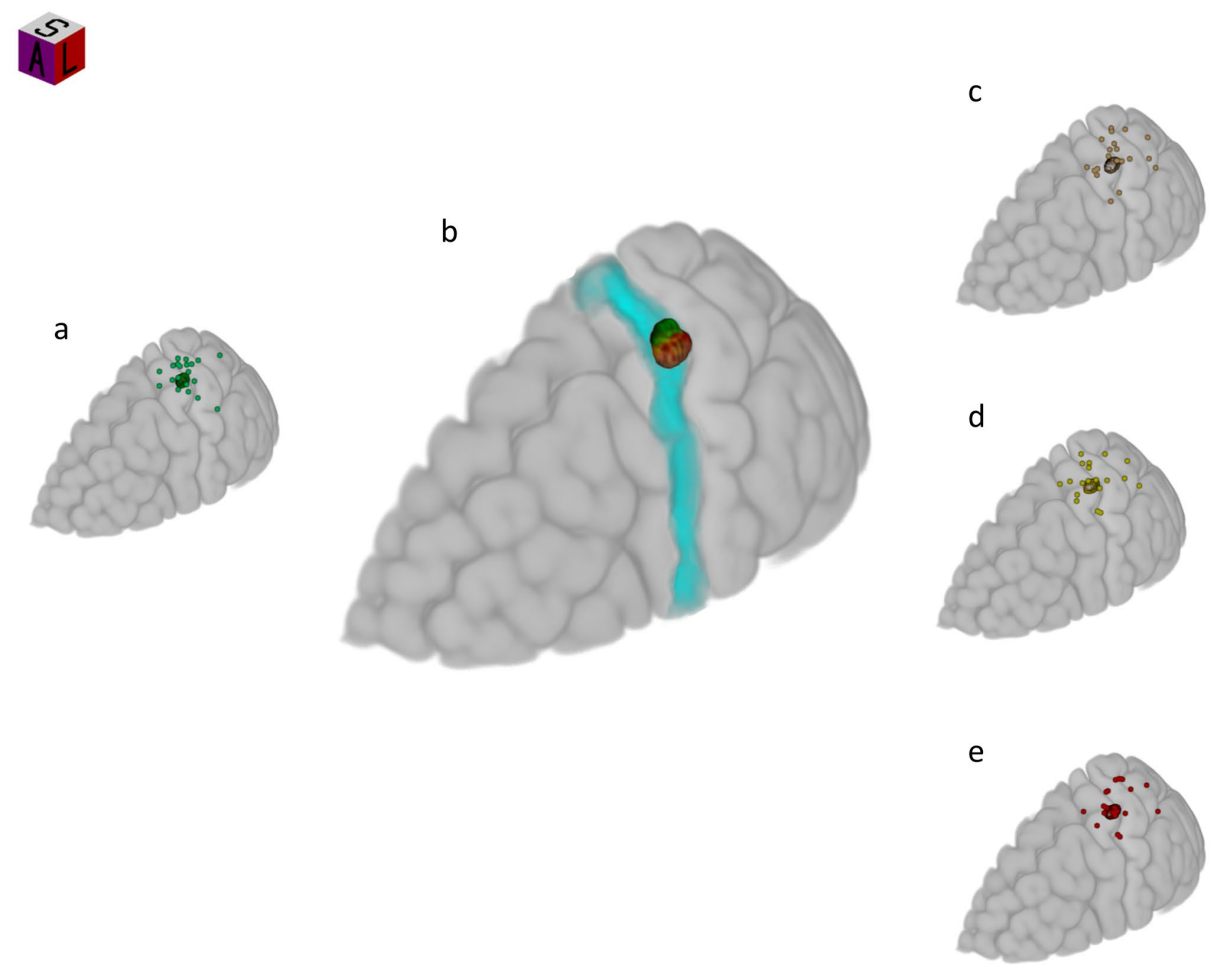
Individual CoG superimposed on Colins 27 MNI-brain for cMEP hotspot (**a**, green), and iMEPs during unilateral (**c**, brown), bilateral homologous (**d**, yellow) and bilateral antagonistic (**e**, red) voluntary contraction. Group mean (95% confidence limits) CoG for each condition superimposed on Colins 27 MNI-brain with a light blue representation of the primary motor cortex M1 (**b**)

**Fig. 3:**
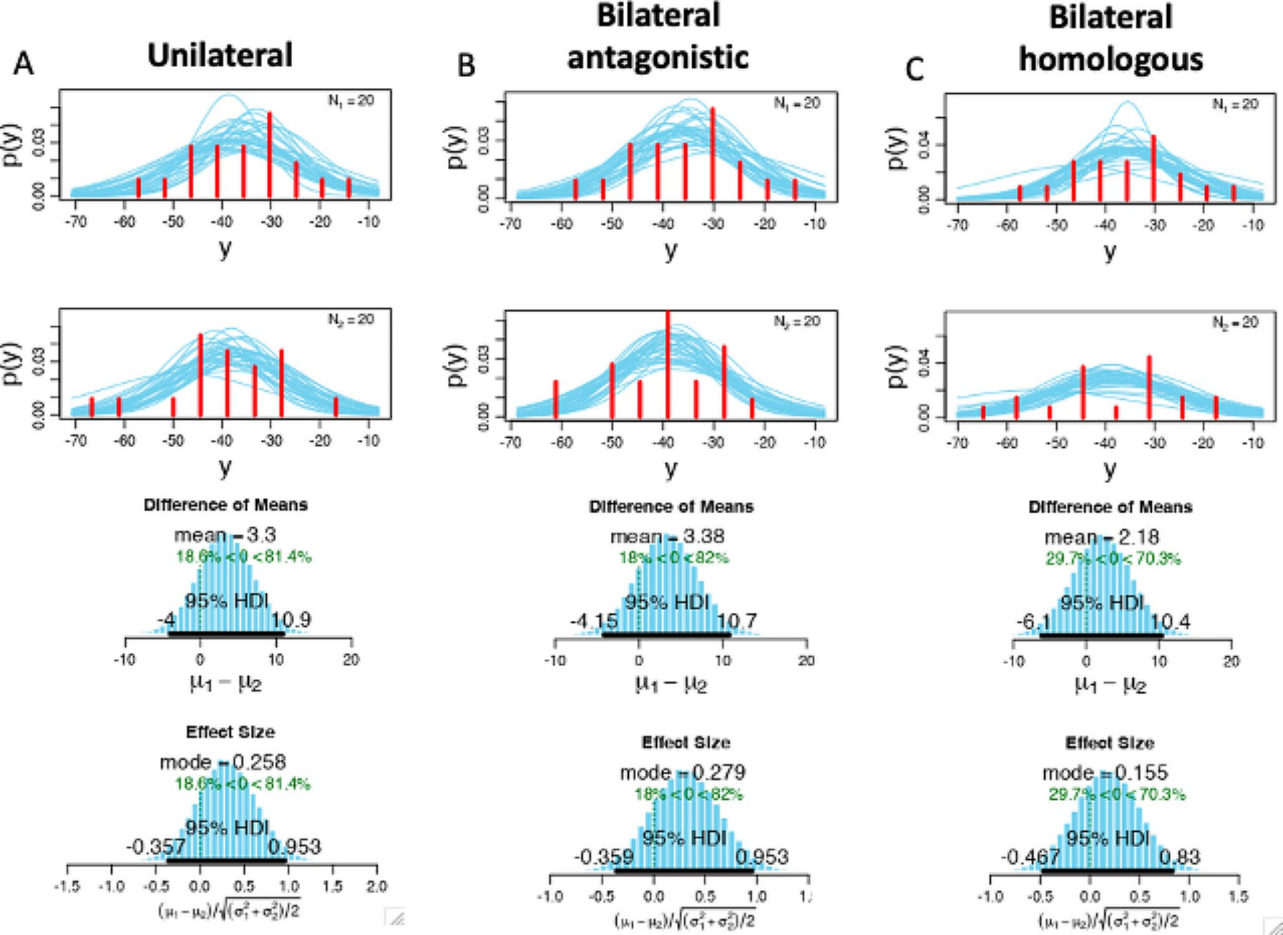
Bayesian statistics for the comparison of the centroids between the three conditions are shown separately for (**a**) iMEP unilateral (**b**) iMEP_bilateral antagonistic (**c**) iMEP_homonym. The first and the second row shows the distribution for the subjects for iMEP and cMEP separately, the third row displays the difference of means for the three conditions separately. P(y) illustrates the probability of the coordinate y. The sufficient effect size is shown in the last row with the 95% highest density interval (HDI) within the data for each comparison separately

Both cMEP hotspot and iMEP CoGs were located around the handknob of the precentral gyrus. However, the center of gravity for iMEPs across all conditions displayed an antero-lateral shift from the cMEP hotspot (MNI coordinates x=-34.2, y=-37.1, z = 63.6) to the iMEP CoGs (see Fig. 2).The comparison of each iMEP CoG and cMEP hotspot separately showed significant differences for iBB (81.4% = *p* < 0.05) shown in Fig. 3a, and iBB-cAE (82% = *p* < 0.05; Fig. 3b) but not bBB (70.3% = *p* > 0.05; Fig. 3c) compared to cMEP hotspot, with a sufficient effect size for each comparison.

**Fig. 4:**
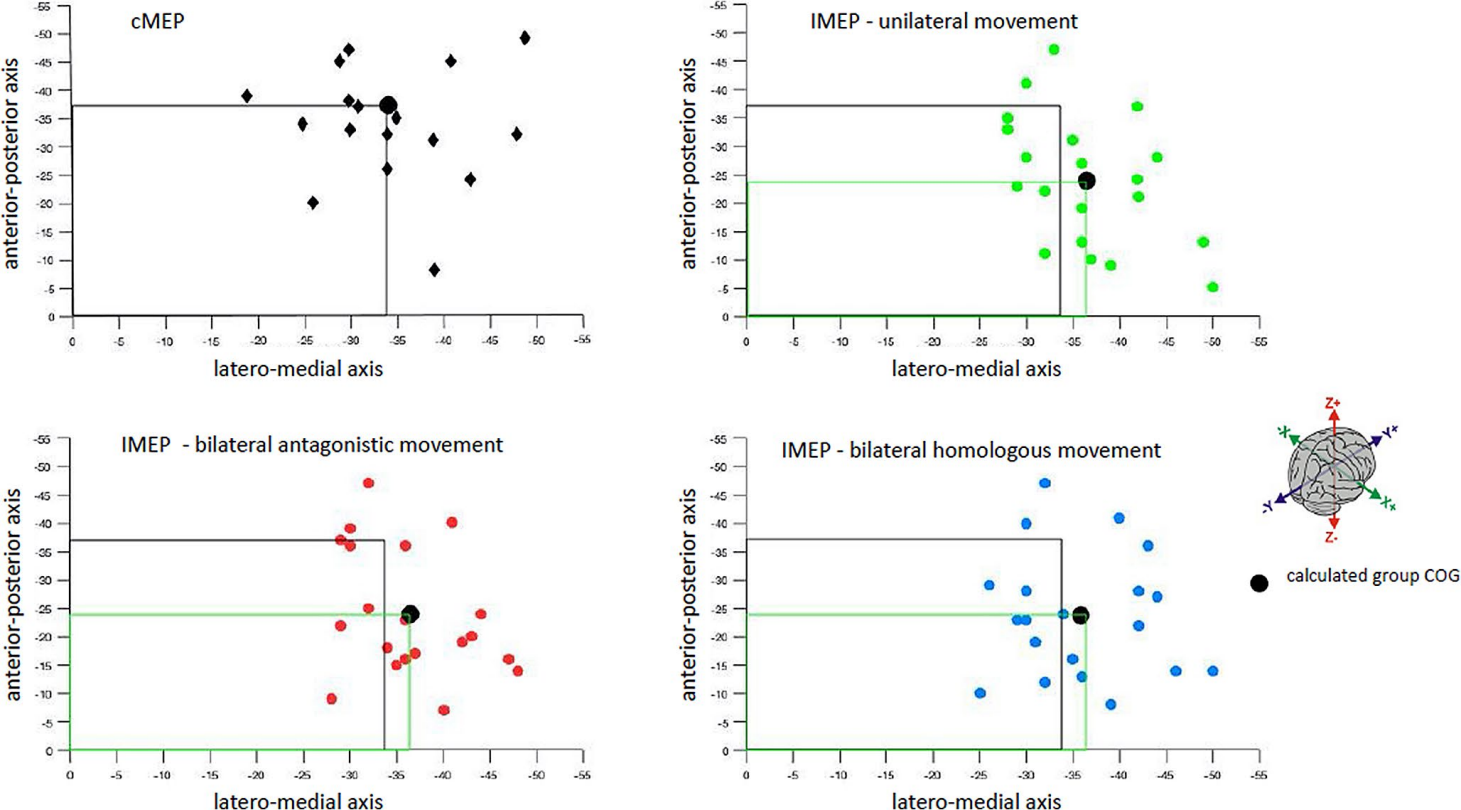
Scatter plot of individual and mean center of gravity (CoG) location for each iMEP compared to cMEP hotspot. The black line to the y- and x-axis in all plots shows the cMEP hotspot location for comparison

Further examination of task-dependent iMEP CoGs (MNI coordinates: iBB − 36.5, -23.85, 63.3; bBB − 35.8, -23.7, 64.3; iBB-cAE − 36.5, -24, 63.85) revealed consistency across the three conditions, as visually depicted in the scatterplot (Fig. 4).

### IMEP measurements

IMEPs in left biceps brachii could be elicited in all participants during all tasks. Levene’s test revealed homogeneity of variance. The iMEP area, expressing the relative size of iMEP compared to the prestimulus EMG, were comparable between tasks: LMM revealed no significant effect of TASK on iMEP area, neither at the target points with the maximum area (F = 0.01, *p* = 0.93) nor at the cMEP hotspots (F = 0.68, *p* = 0.51). In detail iMEP area was 346.6 ± 310.8% for unilateral, 310.9 ± 232.7% for bilateral homologous and 264% ± 128.6% for bilateral antagonistic movement at the target points with the maximum area, and 185.1%±49.4% for the unilateral, 180.1%±63.43% for bilateral homologous and 188.4%±59.17% for bilateral antagonistic movement at the hotspot of the contralateral biceps brachii.

At the target points with the maximum area, mean iMEP onset was 19.8 ± 2.5 ms for iBB, 20.4 ± 3.9 ms for bBB and 20.8 ± 3.1 ms for iBB-cAE, and mean iMEP duration was 23.9 ± 15.2 ms for iBB, 21.3 ± 13.7 ms for bBB and 25.1 ± 16.6 ms for iBB-cAE.

In addition to the lack of variance in area, onset and duration of iMEPS, persistence of iMEPs remained consistent across the different tasks.

## Discussion

Main finding of this investigation is that ipsilateral BB activation, with or without contralateral extension, leads to a shift in the ipsilateral CoG anterolaterally relative to the hotspot of the contralateral BB at rest. Our findings also suggest that both iMEP CoGs and iMEP areas do not vary between unilateral, bilateral homologous, and bilateral antagonistic contractions.

### Location of centre of gravity

The difference on map CoG indicates a shift in motor cortex excitability away from the point from which the same muscle can be activated in relaxation.

This finding agrees with earlier research reporting a different origin for iMEPs compared to cMEPs, but with partly contradictory results for different muscles so far. An antero-lateral shift was reported for iMEP in FDI (Wassermann et al. 1994; Ziemann et al. 1999), whereas an antero-medial shift was reported for the same target muscle as in our study, the biceps brachii, by Tazoe and Perez (Tazoe and Perez 2014). For more proximal muscles like the latissimus dorsi and the pectoralis major, no difference (latissimus dorsi) or a more posterior location (pectoralis major) was reported for iMEPs (MacKinnon et al. 2004), suggesting that differential excitability might also vary between the target muscles. Furthermore, Chen et al. demonstrated that contralateral and ipsilateral MEPs in FDI are preferentially elicited with different current directions over the primary motor cortex (Chen et al. 2003), again suggesting a spatial distinct excitability.

Both iMEP CoGs and cMEP hotspots were located within Brodman area (BA) 4, indicative of the corticospinal tract involved in the production of voluntary movement (Chouinard and Paus 2006; Geyer et al. 1996). The CoG of the cMEPs was located more medio-posterior compared to the more antero-lateral CoGs of the iMEPs, but only CoGs for antagonistic and unilateral, but not homologous tasks were significantly differing from the contralateral BB CoG. Increasing demand during upper limb motor tasks is associated with a differential activation of ipsilateral motor areas (Buetefisch et al. 2014). If this difference is also leading to the task-dependent difference in CoGs in our study remains unclear, and has to be investigated in further studies on proximal upper limb movements. We are also aware of the overall small, albeit significant, difference of our findings, which again emphasizes the need for additional studies, especially since ipsilateral motor pathways are potential target regions for neuromodulation in diseases like stroke (Bradnam et al.^2013^).

Adjacent to BA4 in the antero-lateral spatial direction is the dorsal premotor cortex (PMd) (Geyer et al. 2019; Sigl et al. 2019). The ipsilateral dorsal premotor cortex is involved in self-paced movements (Huang et al. 2004), and especially imaging studies suggests that the caudal part of the PMd plays an important role in motor-coordination and execution of movements (Genon et al. 2017, 2018; Horenstein et al. 2009). In addition, the PMd is involved in modulating the ipsilateral M1 both in an inhibitory and facilitating manner, probably via cortico-cortical connections (Côté et al. 2017; Groppa et al. 2012). Interestingly, after stroke single-pulse TMS over the PMd may give rise to iMEPs of the ipsilateral (unaffected) FDI (Alagona et al. 2001). Such iMEPs during PMd stimulation are seldom observed with healthy participants. These iMEPs may therefore result from an up-regulation of ipsilateral descending pathways after injury to the contralateral corticomotoneural tract.

In sum, our results indicate that the activation of the ipsilateral biceps brachii alters the involved motor network within the primary motor area at the border to the ipsilateral dorsal premotor area, which further supports the role of the ipsilateral hemisphere for motor execution (Bundy and Leuthardt 2019). This is of great interest, as it implies a potential goal for targeted, non-invasive brain stimulation, but also emphasizes task-specific network modulation even under healthy conditions.

### Modulation of iMEP

In our study, the CoG location differed at least in part with the contralateral motor hotspot, but the iMEP area size were independent of task modulation. Therefore, we could not confirm our hypothesis that the cortical iMEP representation is task-dependent. This was somewhat unexpected, as Tazoe and Perez (2014) demonstrated a task dependency for the same upper limb conditions compared to our study. Only a few studies also investigated iMEP task dependency. Another study did not observe differences in iMEP amplitudes in the erector spinae during a unilateral vs. bilateral tonic contraction (Jean-Charles et al. 2017). Bawa et al. (2004) investigated ipsilateral MEPs in proximal and distal muscles during rest, phasic or tonic contraction, and demonstrated that iMEPs in BB were only detectable during phasic, but not tonic bilateral contraction. Methodological differences between the studies might explain the different results to a certain extent, as we here used MR-navigated TMS in a larger sample with more stimuli (10 stimuli per target with in sum 90 stimuli per condition) with 100% MSO as stimulus intensity. Additionally, TMS as well as functional imaging studies reveal complex interaction between bilateral motor and premotor cortices during unilateral vs. bilateral movements (Stinear and Byblow 2002; Walsh et al. 2008), and even unilateral movements relies on bilateral networks (Beaule et al. 2012). Our data with iMEP CoGs and areas not depending on the task suggest that at least the cortical excitability is not altered during different tasks. Together with the partly contradictory results about the modulatory influence of interhemispheric inhibition (Jean-Charles et al. 2017; Perez et al. 2014) and neck afferent inputs (Tazoe and Perez 2014), the need for further studies with different methodological approaches becomes clear.

### Limitations

The present study has limitations. The sample size of 20, although relatively large for an iMEP study, may have been too small to detect task-dependent effects. We were able to demonstrate a presence of iMEPs in each participant, but with large interindividual variance. Further investigations might help to reduce this variance, for example using stimulus intensities adapted to motor thresholds rather than fixed protocols, smaller coils with a more focused area of stimulation, more stimuli and larger group size. There were other methodological limitations. Both the calculation of the individual CoG per task as well as the normalization process for analyzing the data may result in a loss of spatial resolution. Additionally, the 2 cm spacing within the grip, and the high stimulus intensity might also limit the spatial resolution, although they do not alter CoG reliability (Littmann et al. 2013; van de Ruit and Grey 2016). Furthermore, we only assessed CoG for iMEP measurements, and compared it to the cMEP hotspot due to the extensive protocol. Therefore, we cannot explicitly exclude out a spatial shift of the cMEP hotspot. Additionally, as we only tested iMEPs during isotonic contraction, we cannot explicitly exclude that the contraction only primed the ipsilateral motor pathways.

On balance, our MR-navigated approach to obtain iMEPs represents and advance over previous studies to date. Additional methodological approaches may be needed to further enhance our knowledge of the distinct underlying pathways and their modulation during different motor tasks.

## Conclusion

Our findings indicate potential distinctions in the involvement of motor networks within the motor cortex for ipsilateral and contralateral MEPs in a task-specific manner.

## Data Availability

The datasets generated and analyzed during the current study are available from the corresponding author on reasonable request.

## References

[CR1] Alagona G (2001). Ipsilateral motor responses to focal transcranial magnetic stimulation in healthy subjects and acute-stroke patients. Stroke.

[CR2] Armand J, Kuypers HG (1980). Cells of origin of crossed and uncrossed corticospinal fibers in the cat: a quantitative horseradish peroxidase study. Exp Brain Res.

[CR4] Bawa P (2004). Bilateral responses of upper limb muscles to transcranial magnetic stimulation in human subjects. Exp Brain Res.

[CR5] Beaule V, Tremblay S, Theoret H (2012). Interhemispheric control of unilateral movement. Neural Plast.

[CR3] Bradnam LV, Stinear CM, Byblow WD (2013). Ipsilateral motor pathways after stroke: implications for non-invasive brain stimulation. Front Hum Neurosci.

[CR6] Bundy DT, Leuthardt EC (2019). The cortical physiology of Ipsilateral Limb movements. Trends Neurosci.

[CR7] Cavaleri R, Schabrun SM, Chipchase LS (2017). The number of stimuli required to reliably assess corticomotor excitability and primary motor cortical representations using transcranial magnetic stimulation (TMS): a systematic review and meta-analysis. Syst Rev.

[CR100] Butefisch CM (2000) Mechanisms of use-dependent plasticity in the human motor cortex. Proc Natl Acad Sci 97(7):3661–3665. 10.1073/pnas.97.7.3661 10.1073/pnas.050350297 PMC16296 10716702

[CR8] Chen R, Yung D, Li JY (2003). Organization of ipsilateral excitatory and inhibitory pathways in the human motor cortex. J Neurophysiol.

[CR9] Chouinard PA, Paus T (2006). The primary motor and premotor areas of the human cerebral cortex. Neuroscientist.

[CR10] Côté SL (2017). Contrasting Modulatory effects from the dorsal and ventral Premotor Cortex on Primary Motor Cortex outputs. J Neurosci.

[CR11] Genon S (2017). The right dorsal Premotor Mosaic: Organization, functions, and Connectivity. Cereb Cortex.

[CR12] Genon S (2018). The heterogeneity of the left dorsal premotor cortex evidenced by multimodal connectivity-based parcellation and functional characterization. NeuroImage.

[CR13] Geyer S (1996). Two different areas within the primary motor cortex of man. Nature.

[CR14] Geyer S, Ledberg A, Schleicher A, Kinomura S, Schormann T, Bürgel U, Klingberg T, Larsson J, Zilles K, Roland P (2019) Probabilistic cytoarchitectonic map of area 4a (PreCG) (v9.4). Human Brain Project Neuroinformatics Platform

[CR15] Groppa S (2012). The human dorsal premotor cortex facilitates the excitability of ipsilateral primary motor cortex via a short latency cortico-cortical route. Hum Brain Mapp.

[CR16] Hanuscheck N et al (2022) Interleukin-4 receptor signaling modulates neuronal network activity. J Exp Med 219 10.1084/jem.20211887 PMC9123307 35587822

[CR17] Horenstein C (2009). Comparison of unilateral and bilateral complex finger tapping-related activation in premotor and primary motor cortex. Hum Brain Mapp.

[CR18] Huang MX (2004). Temporal dynamics of ipsilateral and contralateral motor activity during voluntary finger movement. Hum Brain Mapp.

[CR19] Jean-Charles L (2017). Interhemispheric interactions between trunk muscle representations of the primary motor cortex. J Neurophysiol.

[CR20] Johnson VE (2013). Revised standards for statistical evidence. Proc Natl Acad Sci U S A.

[CR21] Kruschke JK (2013). Bayesian estimation supersedes the t test. J Exp Psychol Gen.

[CR22] Kruschke JK (2021). Bayesian analysis reporting guidelines. Nat Hum Behav.

[CR23] Littmann AE, McHenry CL, Shields RK (2013). Variability of motor cortical excitability using a novel mapping procedure. J Neurosci Methods.

[CR24] Lotze M (2003). Comparison of representational maps using functional magnetic resonance imaging and transcranial magnetic stimulation. Clin Neurophysiol.

[CR25] MacKinnon CD, Quartarone A, Rothwell JC (2004). Inter-hemispheric asymmetry of ipsilateral corticofugal projections to proximal muscles in humans. Exp Brain Res.

[CR26] McCambridge AB, Stinear JW, Byblow WD (2016). Are ipsilateral motor evoked potentials subject to intracortical inhibition?. J Neurophysiol.

[CR27] Michels L (2017). Changes of Functional and Directed Resting-State Connectivity Are Associated with neuronal oscillations, ApoE genotype and amyloid deposition in mild cognitive impairment. Front Aging Neurosci.

[CR28] Miranda PC (1997). A new method for reproducible coil positioning in transcranial magnetic stimulation mapping. Electroencephalogr Clin Neurophysiol.

[CR29] Oldfield RC (1971). The assessment and analysis of handedness: the Edinburgh inventory. Neuropsychologia.

[CR30] Perez MA, Butler JE, Taylor JL (2014). Modulation of transcallosal inhibition by bilateral activation of agonist and antagonist proximal arm muscles. J Neurophysiol.

[CR31] Sigl B, Caspers S, Bludau S, Mohlberg H, Eickhoff SB, Amunts K (2019) Probabilistic cytoarchitectonic map of Area 6d1 (PreCG) (v4.1). Human Brain Project Neuroinformatics Platform

[CR101] Staudt M, Grodd W, Gerloff C, Erb M, Stitz J, Krägeloh?Mann I (2002) Two types of ipsilateral reorganization in congenital hemiparesis: a TMS and fMRI study. Brain 125(10), 2222–2237. 10.1093/brain/awf227 10.1093/brain/awf227 12244080

[CR32] Stinear JW, Byblow WD (2002). Disinhibition in the human motor cortex is enhanced by synchronous upper limb movements. J Physiol.

[CR33] Tazoe T, Perez MA (2014). Selective activation of ipsilateral motor pathways in intact humans. J Neurosci.

[CR34] van de Ruit M, Grey MJ (2016). The TMS map scales with increased stimulation intensity and muscle activation. Brain Topogr.

[CR35] Walsh RR (2008). Network activation during bimanual movements in humans. NeuroImage.

[CR36] Wassermann EM, Pascual-Leone A, Hallett M (1994). Cortical motor representation of the ipsilateral hand and arm. Exp Brain Res.

[CR37] Yousry TA (1997). Localization of the motor hand area to a knob on the precentral gyrus. A new landmark. Brain.

[CR38] Zdunczyk A (2013). The reliability of topographic measurements from navigated transcranial magnetic stimulation in healthy volunteers and tumor patients. Acta Neurochir (Wien).

[CR39] Ziemann U (1999). Dissociation of the pathways mediating ipsilateral and contralateral motor-evoked potentials in human hand and arm muscles. J Physiol.

